# High Dose Vitamin B6 Reduces Depression Symptoms

**DOI:** 10.1155/da/8926512

**Published:** 2026-08-07

**Authors:** David T. Field, Rebekah Cracknell, Nicolo Biagi

**Affiliations:** ^1^ School of Psychology and Clinical Language Sciences, The University of Reading, Reading, Berkshire, UK, reading.ac.uk

**Keywords:** anxiety, depression, inflammation, pyridoxine, stress, vitamin B6

## Abstract

**Background:**

Several lines of evidence suggest that vitamin B6 has potential as an antidepressant. It plays a crucial coenzyme role in the synthesis of the neurotransmitters serotonin, gamma‐aminobutyric acid (GABA) and dopamine, which are central to mood regulation, and it has been shown that increasing availably of vitamin B6 above normal levels increases concentrations of serotonin and GABA. Furthermore, epidemiological evidence points to vitamin B6 deficiency as a risk factor for depression, and to high vitamin B6 levels as protective. Therefore, we asked whether taking 100 mg vitamin B6 as pyridoxine daily for 1 month would reduce depression symptoms, measured with the Depression, Anxiety and Stress Scales (DASS) questionnaire, which also assesses anxiety and stress.

**Methods:**

We recruited 267 participants from the general population (215 female) and double blind randomised them to receive either placebo, vitamin B6 or vitamin B12 (1000 µg). Using baseline DASS scores, we divided participants into a group classified as having mild or more severe depression symptoms at baseline (*N* = 58) and a larger group classified as non‐depressed.

**Results:**

Only within the former group, vitamin B6 produced a selective reduction in depression symptoms compared to placebo or vitamin B12 with a very large effect size (partial *η* ^2^ = 0.20). We also found evidence that participants whose DASS depression scores narrowly failed to meet criterion for moderate depression (*N* = 52) experienced a slightly smaller but still statistically substantial reduction in symptoms (partial *η* ^2^ = 0.17). We repeated the analysis procedure for anxiety and stress scores but found no significant effects.

**Conclusions:**

We have shown that high‐dose vitamin B6 has therapeutic potential for depression, and large‐scale clinical trials with patients should now be conducted to determine whether these findings from our mostly female sample generalise to males, to quantify the dose–response relationship, and to evaluate its potential as an adjunct to existing treatments versus monotherapy, and to elucidate which factors, for example, presence of inflammation, predict responsiveness to the intervention.

## 1. Introduction

Negative emotional states and mental health challenges such as depression, anxiety and stress have high prevalence, resulting in great societal and economic cost [1–3], and a high proportion of cases go untreated [4]. Existing drug treatments are not effective for many patients, with a recent analysis of data from over 70,000 clinical trial participants finding that only 15% of patients experience a substantial benefit beyond placebo [5]. This suggests a need to develop alternative or adjunctive interventions that avoid the drawbacks of existing treatments, which include side effects and social stigma in the case of psychotropic medications, and the difficulty of rolling treatment out at a sufficient scale in the case of talking therapies such as cognitive behavioural therapy and counselling [6–8].

One potential intervention for depression, anxiety and stress is high‐dose vitamin B6, which performs a crucial coenzyme role in the synthesis of multiple neurotransmitters in the brain. It has long been known that the active form of vitamin B6, pyridoxal‐5′‐phosphate (PLP), acts as a cofactor for enzymes involved in the biosynthesis of gamma‐aminobutyric acid (GABA), serotonin and dopamine, all of which are central to mood regulation and stress response [9]. If, under normal physiological conditions, the proportion of the relevant enzymes that are not bound to PLP and, therefore, are inactive is significant, then increasing PLP concentration has potential to increase the proportion of active enzyme that is bound to PLP, consequently altering neurotransmitter concentrations. In the case of glutamate decarboxylase (GAD), the enzyme that converts glutamate to GABA, a high proportion of the enzyme exists in the unbound form is well established [10, 11]. Turning to aromatic L‐amino acid decarboxylase (AADC), which converts L‐DOPA to dopamine and 5‐hydroxytryptophan to serotonin, one study using rats estimated that 22% of AADC in the brain exists in the unbound form, and established that saturating a tissue extract with PLP converted all of this to the active bound form [12].

Proof of concept for the proposal that high dose vitamin B6 alters neurotransmitter levels and that this has relevant behavioural consequences is provided by animal models. In one study, rats were fed either a vitamin B6‐deficient diet or a high vitamin B6 diet, and the brains of the latter group were found to contain higher GABA and lower glutamate [13]. While this difference might be explained either by the effect of vitamin B6 deficiency, or of intake exceeding normal dietary levels, or by both of those, a recent study in mice confirmed that exceeding normal dietary levels increases GABA and reduces glutamate [14]. Furthermore, at the behavioural level, anxious behaviour was reduced, and an ‘n’ shaped dose response curve for both this and the effect on GABA/glutamate was established. Turning to serotonin and depression, a recent mouse model of depression revealed an antidepressant effect of vitamin B6, as well as increased serotonin, which was comparable to fluoxetine [15]. Interestingly, the effective dose was lower than the dose previously found to be effective for anxiety by the same group [14]. Turning to rhesus monkeys, a positron emission tomography study found that intravenous infusion with vitamin B6 increased activity of AADC, resulting in 20% increase in the rate of serotonin formation in the corpus striatum, but with noteworthy variation between individual monkeys [16].

Evidence that vitamin B6 status is linked to depression, anxiety and stress in humans has been growing for some time and is most convincing for depression. Cross sectional and longitudinal general population studies, some of which analysed data from very large samples and controlled for multiple confounders, have found that low intake of vitamin B6 or low plasma PLP increases risk of depression and also that high vitamin B6 intake or higher plasma PLP is protective [17–22]. We are aware of two studies that compared the PLP status of depressed patients to controls or standard reference values for vitamin B6 deficiency, and they both found that the rate of PLP deficiency in the depressed group was about three times higher than in the general population [23, 24]. Surprisingly, in light of this evidence, no well‐powered studies of the effects of vitamin B6 supplementation on depression symptoms have previously been reported.

There is also some evidence for a link between anxiety and vitamin B6 intake or PLP status in humans. In a cross‐sectional study that controlled for various potential confounders, being in the bottom tertile of vitamin B6 intake was associated with higher risk of anxiety, particularly in women [25]. Another study compared serum vitamin B6 levels of patients who attended hospital emergency rooms due to panic attack or hyperventilation with controls, finding lower B6 in the anxious group [26]. Turning to intervention studies, we have previously reported that taking 100 mg vitamin B6 for 1 month reduced scores on the Screen for Adult Anxiety Related Disorders (SCAARED) compared to placebo in a sample drawn mainly from a student population with relatively high baseline anxiety scores [27]. This effect was mainly driven by improvement in the Generalised Anxiety Disorder (GAD) subscale. The same study provided evidence that vitamin B6 increased neural inhibition (GABA) relative to excitation, which was suggested by the finding that vitamin B6 increased visual surround suppression. However, two previous intervention studies using non‐clinical samples have found no effect of vitamin B6 supplementation on anxiety or mood, although both of these found beneficial effects on long‐term memory. In one case, the dose used was relatively low (20 mg), which could explain the lack of effect [28]. In the other, the dose was somewhat higher (75 mg), but average anxiety scores of participants at baseline were relatively low compared to those in our study [27], which would make it hard to demonstrate a reduction in symptoms due to vitamin B6 supplementation if, as seems likely, this only occurs in individuals with significant symptoms [29].

Most of the evidence of a reduction by vitamin B6 of psychological stress is indirect and suggestive only and comes from studies of the effects of B‐multivitamins or yeast‐based spreads that are rich in B‐vitamins. However, one found a specific benefit of vitamin B6 in a group of participants selected to be magnesium deficient; B6 provided an additional benefit beyond that from supplementing with magnesium, but only in individuals with very high baseline stress levels [30].

In this paper, we evaluate whether taking a high, pharmacological, dose of vitamin B6 for 1 month influences the Depression, Anxiety and Stress subscales of the Depression, Anxiety and Stress Scales (DASS) questionnaire, in a young adult (mainly student) population. We hypothesised that positive effects would only occur in individuals reporting some depression, anxiety or stress, at baseline, and to test this, we divided participants into groups using the published DASS cutoff scores for mild or greater symptoms. The data analysed here were collected from the same participants as that reported in our previous paper reporting positive effects of vitamin B6 on SCAARED anxiety scores, as well as increased visual surround suppression, and formed part of a larger test battery described in detail in our previous publication [27]. The test battery was constructed to evaluate the broader hypothesis that high dose vitamin B6 would alter the neural excitation–inhibition balance (EIB), and therefore included a range of measures that have previously been linked to the EIB, including depression [31]. Participants were double‐blind randomised to three groups—placebo, vitamin B6, and vitamin B12, and here, the vitamin B12 participants provide an additional control/comparison group.

## 2. Methods

### 2.1. Participants and Design

Data collection was conducted in the United Kingdom in five phases over 5 years as described in [27]. The DASS questionnaire was included as an outcome measure in phases 2–5, which recruited a total of 325 participants. Of these, 268 (82%) completed the DASS at both baseline and post‐test and form the sample analysed here. For full details of the test battery in each of the five phases of data collection, see Field et al. [27], particularly Section 2.1 and Tables 1 and 2. Note that data collection for phases 1–4 was conducted in person, but due to COVID‐19, this moved online in phase 5. In this phase, bottles of vitamins or placebos were sent to participants in the post, and an online version of the test battery was constructed using the Gorilla Experiment Builder (www.gorilla.sc); see [27] for further details.

**Table 1:** DASS depression scores at baseline and post‐test, as well as mean age and percent of the sample that was female, broken down by group allocation at baseline and treatment.

Baseline group	Intervention	*N*	Mean age	Percent female	Mean depression score at baseline	SD depression score at baseline	Median depression score at baseline	Mean depression score at post‐test	SD depression score at post‐test	Median depression score at post‐test
Depressed	Placebo	18	21.0	94.4	13.9	3.6	13	10.6	7.7	9
Depressed	Vitamin B6	19	22.9	89.5	17.8	6.7	18	8.2	6.4	6
Depressed	Vitamin B12	21	21.5	71.4	15.7	6.0	14	13.9	8.6	13
Not depressed	Placebo	65	22.2	76.9	3.0	2.7	3	4.1	4.1	3
Not depressed	Vitamin B6	62	24.7	75.8	3.2	3.0	2	3.5	4.7	2
Not depressed	Vitamin B12	69	24.9	85.5	2.5	2.5	2	3.8	5.4	2

**Table 2:** DASS anxiety scores at baseline and post‐test, broken down by group allocation at baseline and treatment, as well as mean age and percentage of the sample that was female.

Baseline group	Intervention	*N*	Mean age	Percent female	Mean anxiety score at baseline	SD anxiety score at baseline	Median anxiety score at baseline	Mean anxiety score at post‐test	SD anxiety score at post‐test	Median anxiety score at post‐test
Anxious	Placebo	18	21.8	94.4	10.9	2.7	10	8.8	3.7	9
Anxious	Vitamin B6	21	22.1	90.5	12.6	4.7	11	11.4	6.0	12
Anxious	Vitamin B12	16	21.7	87.5	11.6	3.4	10	10.6	6.7	10
Not anxious	Placebo	65	21.9	76.9	2.4	2.2	2	3.1	3.2	2
Not anxious	Vitamin B6	60	25.1	75.0	2.2	1.9	2	2.8	3.6	2
Not anxious	Vitamin B12	74	24.6	81.1	2.6	2.2	2	3.4	3.8	2

Of the 268 participants analysed here, 215 were female. Ages of participants ranged from 18 to 61, with the distribution skewed towards young adults: the mean age was 23.3 (SD 7.9), but the median was 20. Of these participants, 86 were double‐blind randomised to receive lactose placebo tablets, 88–100 mg vitamin B6 as pyridoxine hydrochloride, and 94–1000 µg vitamin B12 as methylcobalamin. To provide some flexibility in the scheduling of follow‐up lab visits, the period of supplementation was allowed to vary between 30 and 40 days.

To randomise and double‐blind the study, after medicine bottles were filled with tablets, each was numbered, and the contents recorded. Numbers were allocated to bottles randomly using a computerised method by a PhD student from the corresponding author’s lab who was not connected to the study. During the baseline session, each participant retrieved a bottle from a bag containing all the bottles, and the experimenter recorded the number on the bottle. However, in phase 5, when the study was moved online, an experimenter selected at random a sealed envelope containing one of the bottles with the bottle number written on the outside and wrote the participant postal address on it. To encourage compliance, participants were asked to return bottles at the end of the study and were told that experimenters would check how many tablets remained in the bottle.

To take part, participants had to confirm that they were not lactose intolerant, diabetic, epileptic, or taking medications known to interfere with B‐vitamin absorption. They were also asked if they were taking supplements that contained B vitamins and were excluded unless they agreed to stop taking them for the duration of their participation in the study. The study was approved by the University of Reading Ethics Committee (2018‐128‐DF). Written informed consent was obtained from all participants, and an online tick box replaced the requirement for a signature in phase 5 of data collection. No participants reported the known side effects of taking pyridoxine at high doses (tingling sensations in extremities, an early sign of peripheral neuropathy). One participant in the pyridoxine group reported experiencing vivid dreams and withdrew for that reason.

### 2.2. DASS Questionnaire

The DASS‐42 [32] is a 42‐item self‐report instrument designed to measure the negative emotional states of depression, anxiety and stress. Each of the three subscales comprises 14 items, rated on a 4‐point Likert scale ranging from 0 (“Did not apply to me at all”) to 3 (“Applied to me very much or most of the time”). Participants were asked to reflect on their experiences over the past week. Subscale scores were calculated by summing the relevant items, with higher scores indicating greater severity of symptoms. The DASS‐42 has demonstrated strong psychometric properties and is widely used in both clinical and research settings to assess emotional distress [33]. Severity of symptoms is classified according to established cutoffs provided in the original manual: scores of 10 or above on the Depression subscale, 8 or above on the Anxiety subscale, and 15 or above on the Stress subscale indicate mild or greater symptom severity [32]. Cutoffs for moderate, severe and extremely severe symptoms are also provided, but in the current study, we pooled all participants reporting mild or more severe symptoms at baseline into a single group.

### 2.3. Data Analysis

We first flagged participants as outliers if their DASS total score at baseline or post‐test was extreme. Because the distribution of DASS‐total scores was positively skewed, we used the median absolute deviation (MAD) method of flagging outliers, with *k* = 3, to do this [34]. Participants flagged as outliers in this way were excluded from all subsequent analysis of D, A and S scores. Some of the extremely high implausible scores may have been caused by participants ‘straight‐lining’ when filling in the DASS questionnaire, which does not contain reversed items.

For the analysis of Depression scores, we created an independent variable to allocate participants with a DASS‐D baseline score ≥10, suggesting mild or more severe depression symptoms at baseline, into one group, with the rest of the participants allocated to the non‐depressed group. The same procedure was followed for DASS‐Anxiety scores (cutoff ≥ 8) and DASS‐Stress scores (cutoff ≥ 15).

To test for effects of vitamin B6 on the DASS subscales, we calculated change scores between baseline and post‐test, such that positive change scores represented a reduction of symptoms (improvement). The change scores approximated a normal distribution, in contrast to the positively skewed raw scores, which is preferable when using ANOVA. Tablet type (3)  ^∗^ Baseline group (2) ANOVAs were performed on the change scores from each DASS subscale, and the main effect of tablet type and the interaction of tablet type with baseline group were assessed. Where the ANOVA results suggested a selective benefit of vitamin B6 for the group with symptoms at baseline, we quantified the effect size (partial *η* ^2^) by comparing the vitamin B6 group to the other two groups combined using one‐way ANOVA.

If there are differences between groups on the outcome measure at baseline, the analysis of treatment effects on change scores can be confounded. Therefore, whenever ANOVA detected an effect of treatment on change scores, we performed further ANOVAs (and *t*‐tests) on the baseline scores to check for the presence of unintended baseline differences between groups that can sometimes occur despite randomisation of participants to treatment conditions. If these were present, we adjusted for them using ANCOVA to repeat the analysis of change scores, with baseline scores as the covariate. This allowed us to determine the extent to which controlling for baseline differences between treatment groups reduced the effect size of the vitamin B6 intervention. Additionally, the outcomes measured by the DASS are correlated with the age and biological sex of participants, which might therefore differ between the experimental groups we defined using DASS scores. Therefore, if ANOVA detected an effect of treatment on change scores, we performed and additional ANCOVA with age and biological sex as covariates in addition to the baseline score on the outcome measure.

Because we are reporting an exploratory data analysis, we have not performed adjustments for multiple comparisons. All *p* values reported are two‐tailed.

Statistical analyses were initially conducted using IBM SPSS Statistics (version 28; IBM Corp.). Analyses were independently replicated in R (R Core Team) for the purpose of figure generation and to verify the results.

## 3. Results

### 3.1. Outliers

The MAD was 12 for the baseline DASS‐Total scores, and 11 for the post‐test DASS‐total scores. Thirteen participants had scores at one or both timepoints that were much greater than the median; these were flagged as outliers by applying the MAD threshold and excluded, resulting in a final sample size of 254. Two participants were excluded from the placebo condition, seven from the vitamin B6 group and four from the vitamin B12 group.

### 3.2. Baseline and Post‐Test DASS Scores

Tables 1–3 present the means, standard deviations and medians of the DASS depression, anxiety and stress scores at baseline and post‐test. These are each broken down by group allocation at baseline (mild or more severe depression, anxiety, or stress versus not depressed, anxious, or stressed) and treatment group. For each group, we have also included the percentage female and the mean age.

**Table 3:** DASS stress scores at baseline and post‐test, broken down by group allocation at baseline and treatment, as well as mean age and percentage of the sample that was female.

Baseline group	Intervention	*N*	Mean age	Percent female	Mean stress score at baseline	SD stress score at baseline	Median stress score at baseline	Mean stress score at post‐test	SD stress score at post‐test	Median stress score at post‐test
Stressed	Placebo	21	21.8	100.0	21.7	6.0	21	17.8	7.6	18
Stressed	Vitamin B6	22	23.7	81.8	20.5	5.2	20	16.3	8.2	16
Stressed	Vitamin B12	13	21.2	84.6	19.4	4.1	18	16.6	7.8	16
Not stressed	Placebo	62	22.0	74.2	5.5	4.5	4	7.6	6.1	7
Not stressed	Vitamin B6	59	24.5	78.0	7.3	4.3	7	7.1	6.4	6
Not stressed	Vitamin B12	77	24.6	81.8	6.1	4.3	6	7.3	5.9	7

### 3.3. Depression

**Figure 1:**
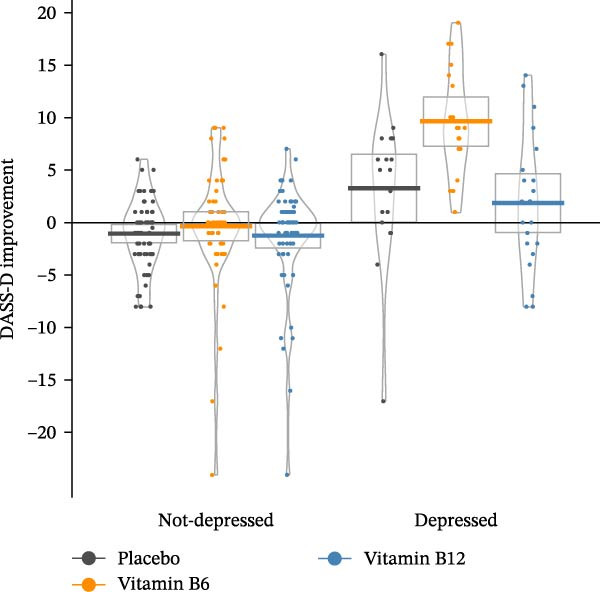
DASS‐depression scores were improved by vitamin B6 compared to placebo or vitamin B12. Mean improvement is plotted separately for participants classified as having mild or more severe depression symptoms at baseline (*N* = 58) and those classified as not depressed (*N* = 196). The graph is a violin plot showing the individual data points and means ± SEM.

Analysis revealed a strong selective reduction of depression symptoms by vitamin B6. Figure 1 presents the DASS‐D change scores in the three treatment groups, broken down to compare the effects on those classified as having mild or more severe depression symptoms at baseline (*N* = 58) to those classified as not depressed (*N* = 196). ANOVA revealed a highly significant main effect of treatment group (*F*(2, 248) = 12.7, *p* < 0.001, partial *η* ^2^ = 0.093), which interacted with depression status at baseline (*F*(2, 248) = 8.1, *p* < 0.001, partial *η* ^2^ = 0.061). To isolate and quantify the effect size of the reduction in depression symptoms caused by vitamin B6 in the group with mild or more severe depression at baseline, we compared change scores in that group (*N* = 19) with change scores in the comparable participants who’d been randomised to receive either placebo or vitamin B12 (*N* = 39). This comparison revealed a very large effect size (partial *η* ^2^ = 0.23), and was highly significant (*F*(1,56) = 17.02, *p* < 0.001).

Inspection of data revealed that the reduction of DASS‐D scores caused by vitamin B6 extended to a further group of participants whose baseline DASS‐D scores were at the higher end of the ‘Normal’ range but did not meet the cutoff for mild depression. To establish this, we compared change scores in participants with DASS‐D scores of 6, 7, 8, or 9 (*N* = 15) at baseline who received vitamin B6, with comparable participants who’d been randomised to receive either placebo or vitamin B12 (*N* = 27). Participants in the vitamin B6 group improved by 4.27 (SD 4.04) points on average, while the comparison group scores declined by −0.89 (SD 6.30). This difference was significant (*F*(1,40) = 8.1, *p* = 0.007), with an effect size that was large (partial *η* ^2^ = 0.17), although smaller than the very large effect size in the group classified as depressed at baseline.

Despite random allocation to conditions, inspection of Table 1 reveals differences between treatment groups in DASS‐D baseline scores, specifically for those participants who were classified as having mild or greater depression at baseline. The baseline DASS‐D scores were higher on average in the vitamin B6 group than in the other groups. This pattern trended towards significance in a 1 ^∗^3 ANOVA comparing baseline scores of participants classified as depressed at baseline across the three treatment groups (*F*(2,55) = 2.20, *p* = 0.120), and a direct comparison by *t*‐test between the placebo and vitamin B6 groups was significant (*t* = 2.18(35), *p* = 0.036). Consequently, the analysis of change scores in the depressed at baseline group presented above may overstate the effect size for the improvement of DASS‐D scores by vitamin B6. This can occur when baseline scores in the treatment group are higher than in the control group and regression to the mean is present in the data, which was the case here, indicated by a positive Pearson correlation between change scores and baseline scores (*r*(254) = 0.47, *p* < 0.001).

Further analysis using ANCOVA to adjust for the baseline differences between treatment groups confirmed that vitamin B6 independently reduced DASS‐D scores. We first conducted ANCOVA to compare DASS‐D change scores between the three treatment conditions, with baseline DASS‐D scores entered as the covariate. The covariate was not significant (*F*(1, 54) = 1.61, *p* = 0.210), while the main effect of treatment group remained highly significant (*F*(2, 54) = 7.36, *p* < 0.001). To re‐quantify the effect size of the reduction in depression symptoms caused by vitamin B6 in the group with mild or more severe depression at baseline while adjusting for the baseline differences, we used ANCOVA to compare change scores in that group (*N* = 19) with change scores in the comparable participants who’d been randomised to receive either placebo or vitamin B12 (*N* = 39). The covariate was not significant (*F*(1, 55) = 1.35, *p* = 0.250), while the effect of treatment was highly significant (1,55) = 14.00, *p* < 0.001), and the effect size remained very large (partial *η* ^2^ = 0.20) but was slightly reduced by including the covariate compared to the effect size in the unadjusted ANOVA above. Confirming the generality of this result, we found the same effect of covariate inclusion—a slight reduction in effect size, with the effect of treatment remaining significant, if the ANCOVA was extended to include the participant subgroup analysed above whose baseline DASS‐D scores were slightly below the clinical cutoff or if it was extended to the entire sample including non‐depressed participants. The only qualitative difference in these two cases was that the covariate was highly significant, and in the case of the whole sample analysis, the effect size of treatment was, predictably, much smaller.

We also used ANCOVAs to further adjust these results for between experimental group differences in age and biological sex (see Table 1). Beginning with the full 3 (treatment group)  ^∗^ 2 (depression status at baseline) model, ANCOVA confirmed the pattern of results found in the unadjusted ANOVA analysis. There was a highly significant main effect of treatment group (*F*(2, 244) = 9.8, *p* < 0.001, partial *η* ^2^ = 0.074), which interacted with depression status at baseline (*F*(1, 244) = 6.3, *p* = 0.002, partial *η* ^2^ = 0.049). However, these effect sizes were reduced by the inclusion of the three covariates, of which baseline DASS‐D score was highly significant (*F*(1, 244) = 10.7, *p* < 0.001, partial *η* ^2^ = 0.042), age trended towards significance (*F*(1, 244) = 2.9, *p* = 0.088, partial *η* ^2^ = 0.012) and biological sex was non‐significant (*F*(1, 244) = 1.0, *p* = 0.324, partial *η* ^2^ = 0.004). Focusing just on those participants classified as reporting mild or more severe depression at baseline, we used ANCOVA with the same three covariates to compare change scores in that group (*N* = 19) with change scores in the comparable participants who’d been randomised to receive either placebo or vitamin B12 (*N* = 39). None of the three covariates was significant (DASS baseline score: *F* (1, 53) = 1.92, *p* = 0.171; biological sex: *F* (1, 53) = 1.90, *p* = 0.174; age *F* (1, 53) = 0.04, *p* = 0.845)), while the effect of treatment was highly significant (1,55) = 11.93, *p* < 0.001), and the effect size remained very large (partial *η* ^2^ = 0.18), but was slightly reduced by including age and biological sex as additional covariates compared to the effect size when only baseline DASS score was adjusted for.

### 3.4. Anxiety

**Figure 2:**
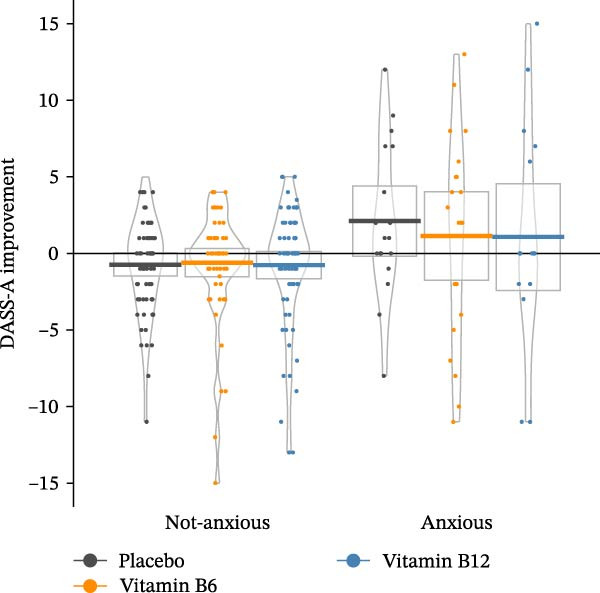
Change in DASS‐anxiety scores in the placebo, vitamin B6 and vitamin B12 groups. Mean improvement is plotted separately for participants classified as having mild or more severe anxiety symptoms at baseline (*N* = 55) and those classified as not anxious (*N* = 199). The graph is a violin plot showing the individual data points and means ± SEM.

Analysis revealed no benefit of vitamin B6 for anxiety symptoms. Figure 2 presents the DASS‐A change scores in the three treatment groups, broken down to compare the effects on those classified as having mild or more severe anxiety symptoms at baseline (*N* = 55) to those classified as not anxious (*N* = 199). ANOVA revealed no main effect of treatment group (*F*(2, 248,) = 0.25, *p* = 0.78, partial *η* ^2^ = 0.002), and no interaction of treatment with group at baseline (*F*(2, 248) = 0.29, *p* = 0.75, partial *η* ^2^ = 0.002).

### 3.5. Stress

**Figure 3:**
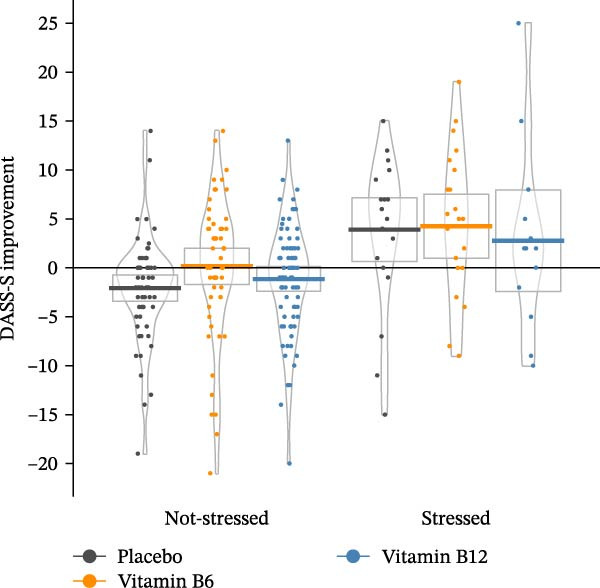
Change in DASS‐stress scores in the placebo, vitamin B6 and vitamin B12 groups. Mean improvement is plotted separately for participants classified as having mild or more severe stress at baseline (*N* = 12) and those classified as not stressed (*N* = 242). The graph is a violin plot showing the individual data points and means ± SEM.

We found no evidence for reduction of stress by vitamin B6. Figure 3 presents the DASS‐S change scores in the three treatment groups, broken down to compare the effects on those classified as experiencing mild or more severe stress at baseline (*N* = 56) to those classified as not stressed (*N* = 198). ANOVA revealed no main effect of treatment group (*F*(2, 248) = 0.86, *p* = 0.42, partial *η* ^2^ = 0.007) and no interaction of treatment with baseline status *F* (2, 248) = 0.46, *p* = 0.63, partial *η* ^2^ = 0.004).

## 4. Discussion

Given existing evidence that high‐dose vitamin B6 influences neurotransmitter levels and that this has behavioural and psychological effects in animals and humans, we explored its effect on three common negative emotional states—depression, anxiety and stress, in an opportunity sample drawn from the general population. Compared to either placebo or high‐dose vitamin B12 tablets, we found that vitamin B6 produced a large improvement in depression symptoms but had no significant effect on anxiety or stress. The largest improvement was evident in participants whose baseline depression scores were classified as indicating mild or more severe depression, but individuals whose baseline depression scores fell just below that cutoff also improved substantially. These effects were confirmed when we adjusted the analysis for baseline differences. This is the first randomised double‐blind study to demonstrate the potential of vitamin B6 as a standalone or adjunctive treatment for depression; it builds on a large body of epidemiological evidence indicating a relationship between vitamin B6 status and depression risk, as well as the previous trials showing benefits of vitamin B6 for the mood/depression symptoms experienced in premenstrual syndrome, and the animal work revealing the potential antidepressant mechanism via increasing serotonin and GABA, and reducing glutamate, in the brain.

Although the role of vitamin B6 in neurotransmitter synthesis described in Section 1 is likely to at least partly underpin the effect on depression observed here, vitamin B6 is involved in about 4% of all enzymatic reactions (~150 reactions), and some of these may also contribute to the effect we have observed. For example, there is growing recognition of the role of vitamin B6 in regulating inflammation [35], and in vitro models using human cell lines demonstrate that it exerts broad spectrum anti‐inflammatory activity [36]. It is well established that there is a bidirectional relationship between depression and inflammation [37, 38], and therefore, the anti‐inflammatory effect of vitamin B6 is potentially therapeutic. Elevated inflammation diverts tryptophan away from serotonin synthesis towards the kynurenine pathway, which results in increased production of inflammatory neurotoxic quinolinic acid, which in turn can overstimulate NMDA receptors and damage GABAergic neurons [39]. Increasing availability of PLP to the kynurenine pathway in the presence of inflammation may be therapeutic because it leads to reduced quinolinic acid production and increases kynurenic acid and anthranilic acid production [40]. Furthermore, vitamin B6 deficiency leads to impaired metabolism of homocysteine, which is inflammatory and neurotoxic when levels are elevated, and this is improved by supplementation [41].

DASS‐Anxiety scores were not influenced by vitamin B6. This appears paradoxical because in the same group of participants anxiety measured using the SCAARED questionnaire was reduced by vitamin B6 [27]. However, the two measures of anxiety differ in various ways, and Pearson’s correlation between DASS‐A and SCAARED total scores among the participants reported here was 0.68, which implies only 47% of variance is shared between the two. A significant point of difference between the two questionnaires is that the DASS‐A does not assess worry, while the SCAARED does, in its GAD subscale. In our previous paper [27], the strongest effect of vitamin B6 was found on the GAD subscale of SCAARED, suggesting that the specific aspect of anxiety reduced by vitamin B6 may be worry. If this is the case, this could explain why we failed to detect an effect of vitamin B6 on DASS‐A scores. In addition to this, we found DASS‐A scores to be positively skewed (towards zero), while SCAARED total and GAD subscales scores were fairly symmetrical around a central tendency. This suggests that SCAARED picks up on more subtle ‘subclinical’ aspects of anxiety that may be more widespread in the population. Consistent with this, the reduction of SCAARED anxiety scores by vitamin B6 reported in our previous paper was relatively small compared to the large reduction of DASS‐depression scores reported here, and unlike the effect on depression, which was concentrated in participants with higher baseline depression scores, the effect was distributed evenly across the range of baseline scores.

Vitamin B6 in pharmacological doses also has therapeutic potential for a number of other conditions apart from depression, which is not surprising given the large number of enzymatic reactions it is involved in, and the implication of differences in neurotransmitter levels and inflammation in a wide range of conditions. For example, low vitamin B6 intake is predictive of migraine [42], and a small‐scale trial found that 80 mg of pyridoxine reduced migraine severity compared to placebo [43]. Therapeutic effects of taking high‐dose vitamin B6 have also been demonstrated for the irritability and mood disorder that occurs in some epilepsy patients as a side effect of taking levetiracetam to control seizures [44] and for premenstrual syndrome [45–47]. More recently, we reported a potential benefit in reducing sensory hyperreactivity, which is a transdiagnostic factor that occurs in many neurodevelopmental conditions [48]. However, it is also important to note that very high doses of vitamin B6 taken in the pyridoxine form for long periods can cause peripheral neuropathy [49]. More recently, it has been reported that in a small proportion of individuals differences in the metabolic processes converting pyridoxine to PLP may cause peripheral neuropathy at much lower doses [50]. Therefore, taking high‐dose vitamin B6 as an intervention should be supervised by medical professionals. Recently, it has been suggested that taking vitamin B6 as pyridoxal (PL) or PLP eliminates the risk of peripheral neuropathy [51], but caution is required until this is confirmed.

This study has a number of limitations that should be addressed in follow‐up studies. We did not collect blood samples and therefore could not confirm participant compliance or that the intervention was successful in raising circulating vitamin B6 levels. However, this does not affect our conclusions because if some participants did not take the tablets, then the effect of this would be to reduce the effect size we report here. The lack of blood samples, or good baseline dietary habit information, also prevented us from testing the prediction arising from the epidemiological evidence we reviewed that effects of vitamin B6 supplementation should be larger in people with low intake or borderline deficiency at baseline. The majority of our participants were female, and the percentage of females was particularly high in the group self‐reporting moderate or greater depression at baseline. Consequently, our findings can be generalised to females but not males because the previous epidemiological evidence pointing to the beneficial role of high circulating levels of vitamin B6 in reducing depression symptoms is also stronger for females than males, further studies are required to ask whether increasing circulating vitamin B6 is beneficial for reducing depression symptoms in males. The study did not recruit from a patient population, and the data collection was exploratory, with multiple interrelated outcomes linked to the neural EIB being measured in addition to those reported here, see [27] for details. Neither did we exclude participants taking antidepressants or ask if they were taking them. For all of these reasons, the findings reported here should be confirmed by a more tightly controlled and preregistered clinical trial.

## 5. Conclusion

In conclusion, we have shown that high‐dose vitamin B6 has potential as a cost‐effective adjunctive treatment for depression or a possibly as a monotherapy. This potential should be formally evaluated by large‐scale clinical trials. Trials should be designed to determine whether patients who respond to the intervention are those with low functional vitamin B6 status at baseline and whether that is in turn caused by the presence of inflammation, which increases the metabolic turnover of vitamin B6. Additionally, trials should determine whether the benefits reported here generalise to males. The dose–response relationship should also be quantified, and this may prove somewhat complex because findings from rodent models of anxiety and depression suggest that increased cortical serotonin and the effects of vitamin B6 on depression may occur at lower doses than changes in GABA and glutamate and anxiety reduction [14, 15].

## Funding

The research was performed as part of the employment of the authors by the University of Reading.

## Conflicts of Interest

The authors declare no conflicts of interest.

## Data Availability

The data that support the findings of this study are available upon request from the corresponding author.
